# Flavagline analog FL3 induces cell cycle arrest in urothelial carcinoma cell of the bladder by inhibiting the Akt/PHB interaction to activate the GADD45α pathway

**DOI:** 10.1186/s13046-018-0695-5

**Published:** 2018-02-07

**Authors:** Gangjun Yuan, Xin Chen, Zhuowei Liu, Wensu Wei, Qinghai Shu, Hussein Abou-Hamdan, Lijuan Jiang, Xiangdong Li, Rixin Chen, Laurent Désaubry, Fangjian Zhou, Dan Xie

**Affiliations:** 1 State Key Laboratory of Oncology in South China; Collaborative Innovation Center for Cancer Medicine, Sun Yat-sen University Cancer Center, Guangzhou, 510060 China; 2 Department of Urology, Sun Yat-sen University Cancer Center, Guangzhou, China; 3 School of Material Science and Engineering, Beijing Institute of Technology, Beijing, China; 4 Therapeutic Innovation Laboratory, UMR7200, CNRS/University of Strasbourg, Strasbourg, France; 5 Sino-French Joint Lab of Food Nutrition/Safety and Medicinal Chemistry, College of Biotechnology, Tianjin University of Science and Technology, Tianjin, China

**Keywords:** FL3, PHB, Urothelial carcinoma of the bladder, GADD45α, Cell cycle

## Abstract

**Background:**

Prohibitin 1 (PHB) is a potential target for the treatment of urothelial carcinoma of the bladder (UCB). FL3 is a newly synthesized agent that inhibits cancer cell proliferation by targeting the PHB protein; however, the effect of FL3 in UCB cells remains unexplored.

**Methods:**

FL3 was identified to be a potent inhibitor of UCB cell viability using CCK-8 (cell counting kit-8) assay. Then a series of in vitro *and* in vivo experiments were conducted to further demonstrate the inhibitory effect of FL3 on UCB cell proliferation and to determine the underlying mechanisms.

**Results:**

FL3 inhibited UCB cell proliferation and growth both in vitro and in vivo*.* By targeting the PHB protein, FL3 inhibited the interaction of Akt and PHB as well as Akt-mediated PHB phosphorylation, which consequently decreases the localization of PHB in the mitochondria. In addition, FL3 treatment resulted in cell cycle arrest in the G2/M phase, and this inhibitory effect of FL3 could be mimicked by knockdown of PHB.

Through the microarray analysis of mRNA expression after FL3 treatment and knockdown of PHB, we found that the mRNA expression of the growth arrest and DNA damage-inducible alpha (*GADD45α*) gene were significantly upregulated. When knocked down the expression of GADD45α, the inhibitory effect of FL3 on cell cycle was rescued, suggesting that FL3-induced cell cycle inhibition is *GADD45α* dependent.

**Conclusion:**

Our data provide that FL3 inhibits the interaction of Akt and PHB, which in turn activates the GADD45α-dependent cell cycle inhibition in the G2/M phase.

**Electronic supplementary material:**

The online version of this article (10.1186/s13046-018-0695-5) contains supplementary material, which is available to authorized users.

## Background

Urothelial carcinoma of the bladder (UCB) is the most common malignancy involving in the genitourinary system, it presents a high risk of recurrence and mortality [1]. Especially, muscle-invasive bladder cancer (MIBC) patients suffer from a poor 5-year survival rate of less than 35% [2]. Adjuvant chemotherapy is helpful to delay the progression of this disease, however, presents limited effects and serious side-effects [3]. Thus, there is an urgent need to develop novel drugs that are both effective and safe.

Prohibitin 1 (PHB) is an evolutionarily conserved adaptor protein involved in diverse biological processes such as transcriptional regulation, cell proliferation, mitochondrial function, and resistance to apoptosis [4, 5]. It is ubiquitously expressed in diverse cellular compartments including the nucleus, cytoplasm and mitochondria [6]. PHB is critical of PHB localization within mitochondria by that PHB involves in and maintains the integrity of mitochondria [7]. Recently, numerous studies have revealed that PHB is largely involved in the pathogenesis of several types of human cancers including prostate, breast, lung and gastric cancers [3, 8–10]. Our previous study also demonstrated that PHB is overexpressed in UCB tissues and correlates with a poor prognosis in UCB patients [3, 11]. Moreover, PHB localization in mitochondria is critical for promoting UCB cell proliferation and this process is in the requirement of Akt-mediated phosphorylation [11]. These data, taken together, suggest that PHB is a potential target for the treatment of UCB.

Flavaglines are natural products that newly isolated from medicinal plants in Asia and were found to have anti-tumor effects in human cancer cell lines without being toxic to healthy cells [12–14]. A recent study reported that Flavaglines target the PHB protein and thus, substantially inhibit cell proliferation in leukemic Jurkat cells by repressing activation of the CRaf-MEK-ERK signaling pathway [12]. Similar effects were observed in several human cancer cell lines such as melanoma, colon cancer, breast cancer, leukemia, and lung cancer [15]. To date, however, the potential inhibitory effect of Flavaglines and its underlying mechanisms in UCB cells remain unexplored.

FL3, a derivative analogue of Flavaglines, is identified to be a potent inhibitor of cancer cell proliferation [16]. To date, however, its biological functions and underlying mechanisms in UCB pathogenesis has not been elucidated. Thus, in the present study, by using a series in vitro and in vivo assays, we investigated the potential inhibitory activity of FL3 in UCB cells and studied if FL3 could also target PHB protein to block the Akt/PHB interaction and consequently influence certain critical signal pathways in UCB proliferation.

## Methods

### Materials

Synthetic PHB ligands and FL3-conjugated compound 6 (an amino linker) were provided from The Laboratory for Therapeutic Innovation, University of Strasbourg (Strasbourg France). All of the drugs were stored at − 20 °C as solids, and were dissolved in dimethylsulfoxide (DMSO) before use.

### Cell culture

UCB cell lines T24, BIU and 5637, and normal bladder uroepithelial cell line SV-HUC-1 were cultured at 37 °C with 5% CO_2_ in RPMI 1640 media supplemented with 10% fetal bovine serum, penicillin (100 U/ml), and streptomycin (100 μg/ml).

### Cell viability assay

Cell viability was determined using the Cell Counting Kit-8 (CCK8) Kit (Dojindo, Kumamoto, Japan). After incubation with concentrations of FL3 and paclitaxel for indicated hours, absorbance was measured at 450 nm according to the manufacture’s introduction. Cell viability was expressed as the percentage of absorbance of cells treated with FL3 or paclitaxel compared with cells treated with DMSO.

### Colony formation

Cells were plated as a density of 500 cells/well in 6-well plates and incubated overnight. Then different concentrations of FL3 were added to each well followed by incubation for 4–5 days. After a growth period, colonies were fixed in methanol for 30 min and stained with 0.1% crystal violet for 1 h.

### Western blot analysis

Cells were harvested and lysed, followed by isolation of the supernatant and measurement of the protein concentration using the bicinchoninic method. Then the protein extracts were separated on 10% sodium dodecyl sulfate polyacrylamide gel electrophoresis (SDS-PAGE) gels, and electrotransferred to a PVDF membrane at 250 mA for 2 h at room temperature. Then, the membrane was blocked in 5% bovine serum albumin, and incubated overnight at at 4 °C with primary antibodies (Akt, phospho-Akt Ser 473, prohibitin-1, phospho (Ser/Thr)-Akt substrate, and cyclin-dependent kinase antibodies were from Cell Signaling Technology, Danvers, MA, USA; REA antibody was from Millipore, Billerica, MA, USA; histone H3, Cox IV, GADD45α, and GAPDH antibodies were from Proteintech Group Inc., Rosemont, IL, USA). The membrane was washed and incubated with secondary antibody for 1 h at room temperature. The signal was measured using the ECL detection system (Tanon, Shanghai, China).

### Immunoprecipitation

Cells (1 **×** 10^8^) were harvested after incubation with 0.5 μM FL3 or DMSO for 24 h. Then the cells were lysed with immunopreciptation (IP) lysate buffer (Beyotime, Shanghai, China) and the protein extracts were isolated. A volume of 20 μl Protein G-agarose beads/tube were washed three times with buffer, followed by incubation with anti-PHB antibody, anti-phospho-Akt antibody or IgG antibody at 4 °C with rotation for 3 h. Equal amounts of protein (21 μg) were incubated with beads overnight at 4 °C. After incubation, The beads were re-suspended in 1× LDS Sample Buffer and boiled for 5 min. Then the proteins were resolved on 10% SDS-PAGE gels for Western blot analysis.

### Generation of the FL3-Affigel

FL3-Affigel beads were generated according to the protocol by Thuaud [16]. Briefly, a volume of 1 ml packed Affilgel 10 (Bio-Rad Laboratories, Hercules, CA, USA) was washed on a glass filter with acetonitrile (ACN) and added to a solution of FL3-conjugated compound 6 (3 mg) in ACN (0.8 ml) and triethylamine (20 μl). This suspension was gently rotated for 12 h at room temperature. Ethanolamine (50 μl) was added and after 4 h, the resin was thoroughly washed with ACN, ethanol and water. Coupled AffiGel-10 agarose beads were stored in PBS containing 0.02% NaN_3_ at 4 °C until further use. PBS was used as the negative control (NC).

### Identification of FL3 binding to PHB protein

T24 cells (1 **×** 10^8^) cells were harvested to obtain total protein extracts. 30 μl FL3- or NC-Affigel beads/tube were incubated with total cell extracts at 4 °C with gentle rotation overnight. After incubation, the bound mixtures were re-suspended in 1× LDS buffer and subjected to SDS-PAGE for Western blot analysis with primary PHB antibody.

### Detection of PHB protein in the subcellular compartments

Approximately 1 **×** 10^8^ cells were harvested after incubation with DMSO (control) or FL3 0.5 μM for 24 h. Using the Nuclear and Cytoplasmic Protein Extraction Kit and Cell Mitochondria Isolation Kit (Beyotime), proteins of the nucleus, cytoplasm, and mitochondria were separately extracted according to the manufacturer’s introductions. Then the extracts were subjected for Western blot analysis.

### Knockdown experiment

The Lenti-Pac HIV Expression Packaging Kit (GeneCopoeia, Rockville, MD, USA) was used to construct stable low-expressing PHB T24 cell lines according to the manufacturer’s protocol. Stable PHB-silenced cells were selected with 0.1% puromycin (Invitrogen, Darmstadt, Germany).

### Apoptosis assay

The percentage of apoptotic cells was determined by using an Annexin V-PE apoptosis detection kit (KeyGen Biotech, Jiangsu, China) according to the manufacture’s introduction. In brief, a total of 10^5^ T24 and BIU cells were harvested after incubation with FL3 for 24 h, then stained these cells with Annexin V and PE subsequently. Finally, the cells were subjected to apoptosis analysis with the ACEA NovoExpress System (ACEA Biosciences Inc., CA, USA).

### Cell cycle analysis

The Cell Cycle Assay Kit (Byeotime) was used to analyze the cell cycle distribution. According to the manufacture’s introduction, FL3-treated or control T24 cells were harvested and dealed with PI, RNase A, Triton X-100 subsequently, followed by cell cycle analysis with the MoFlo XDP Flow Cytometry System (Beckman Coulter, CA, USA).

### Subcutaneous and orthotopic xenografts in Balb/c nude mice

Four-week-old Balb/c nude mice were purchased from Charles River Laboratories (Beijing, China). All of the animal experiments were approved by the Animal Ethics Committee of Sun Yat-sen University (Guangzhou, China). A total of 5 × 10^5^ cells were subcutaneously inoculated into the right flank of mice. When the tumor sizes reached to about 4–6 mm^3^, the mice were randomly divided into four groups with seven mice each, and intraperitoneally injected with FL3 (2 mg/kg, 5 mg/kg), palicetaxel (10 mg/kg, positive control), or DMSO (NC) every 2 days for a total of eight times. Body weights and tumor volume were measured and recorded at the time of administration. At the end of the study, the tumors and main organs including livers, kidneys, lungs, and hearts were removed for further immunohistochemistry experiments. The following formula was used to measure tumor volumes: tumor volume = 1/2 *L* × *W*^2^, where *L* represents length and *W* denotes width.

### Immunohistochemistry

The removed organs and tumors were fixed in formalin and then embedded in paraffin. Sections (4 μm thick) were cut and stained with hematoxylin and eosin (H & E). For further immunohistochemical analysis, sections were de-paraffnized in xylene, hydrated in graded alcohol, and blocked in 3% hydrogen peroxide to inhibit endogenous peroxidase activity. Antigen retrieval was completed by incubating the slides for 5 min in Ethylene Diamine Tetraacetic Acid (EDTA) buffer (pH 8.0). After incubation with 10% goat serum, the slides were incubated with anti-PHB antibody (1:400; Santa Cruz) overnight at 4 °C, followed by incubation with secondary goat anti-rabbit antibody at 37 °C for 30 min. Then, the slides were stained with DAB staining solution for less than 5 min, and re-stained with hematoxylin for 1 min followed by polarization for less than 10 s.

### Statistical analysis

All statistical analyses were performed with IBM SPSS Statistics 19.0 (SPSS Inc., Chicago, IL, USA). All data both in vitro *and* in vivo are presented as mean ± S.D. and assessed by two-detailed Student’s *t*-test with three independent experiments. *P* values of < 0.05 was considered statistically significant.

## Results

### FL3 is a potent inhibitor of UCB cell growth

**Fig. 1:**
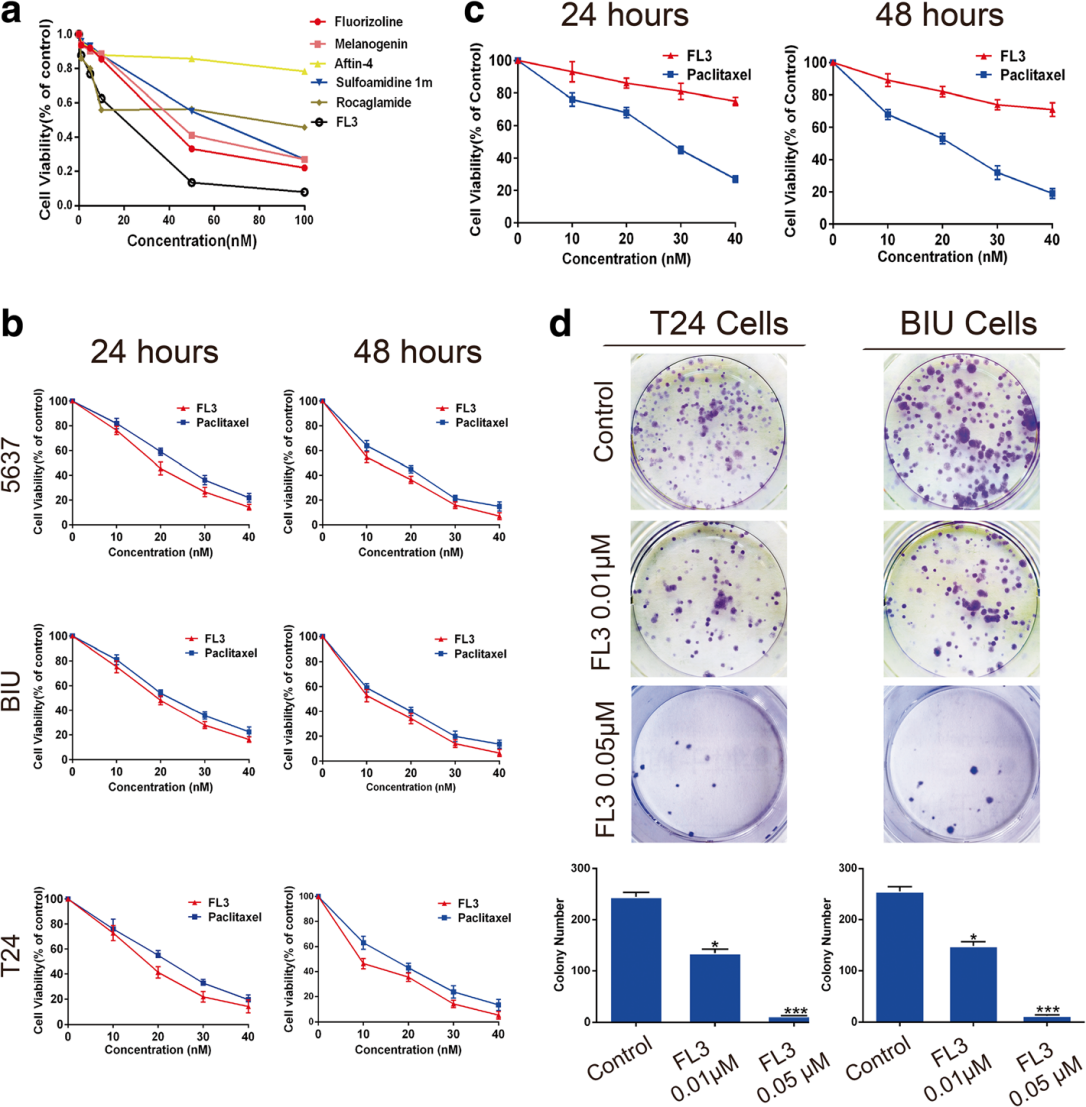
FL3 inhibits the growth and proliferation of UCB cell lines. **a** The CCK-8 assay showed that of the flavaglines tested, FL3 most potently inhibited the cell viability of UCB T24 cells. **b** After incubation with indicated concentrations of FL3 or paclitaxel (positive control) in 5637, T24, and BIU cells for 24 h or 48 h, absorbance of the treated cells was measured at 450 nm. Cell viability was expressed as the percentage of absorbance of cells treated with FL3 or paclitaxel compared with control. **c** CCK-8 assay was performed to examine the cytotoxicity of FL3 and paclitaxel (positive control) to normal bladder uroepithelial SV-HUC-1 cells. **d** Cell colony formation experiments were performed in T24 and BIU cell lines to measure the effects of FL3 on cell proliferation. Histograms display the mean number of colonies, and the number of colonies was shown as the mean ± SD of three independent experiments. *P < 0.05, ***P < 0.001 indicates a significance to control. UCB, urothelial carcinoma of the bladder

To determine if Flavaglines had anti-tumor effects in UCB cells, we measured the cell viability of UCB T24 cells after treatment with various PHB ligands for 24 h. As shown in Fig. 1a, all of PHB ligands used decreased cell viability of T24 cells, in which FL3 exhibited the most potent effect to inhibit cell growth.

### FL3 suppresses UCB cell proliferation in vitro

Next, we explored the inhibitory effect of FL3 on UCB cell proliferation in vitro. To this end, the cell viability assay was performed in three UCB cell lines after FL3 treatment. Paclitaxel, a well-known anticancer agent for some human cancers including UCB, was used as a positive control. As shown in Fig. 1b, FL3 dominantly inhibited the cell viability of three UCB cell lines in a time- and dose-dependent manner. Compared to paclitaxel, FL3 presented less cytotoxicity to normal bladder uroepithelial SV-HUC-1 cells (Fig. 1c). The colony formation assay showed that FL3 treatment significantly inhibited the colony formation ability of T24 and BIU cells compared to the control (Fig. 1d). Taken together, these results showed that FL3 suppressed UCB cell proliferation in vitro.

### FL3 binds to the PHB protein

**Fig. 2:**
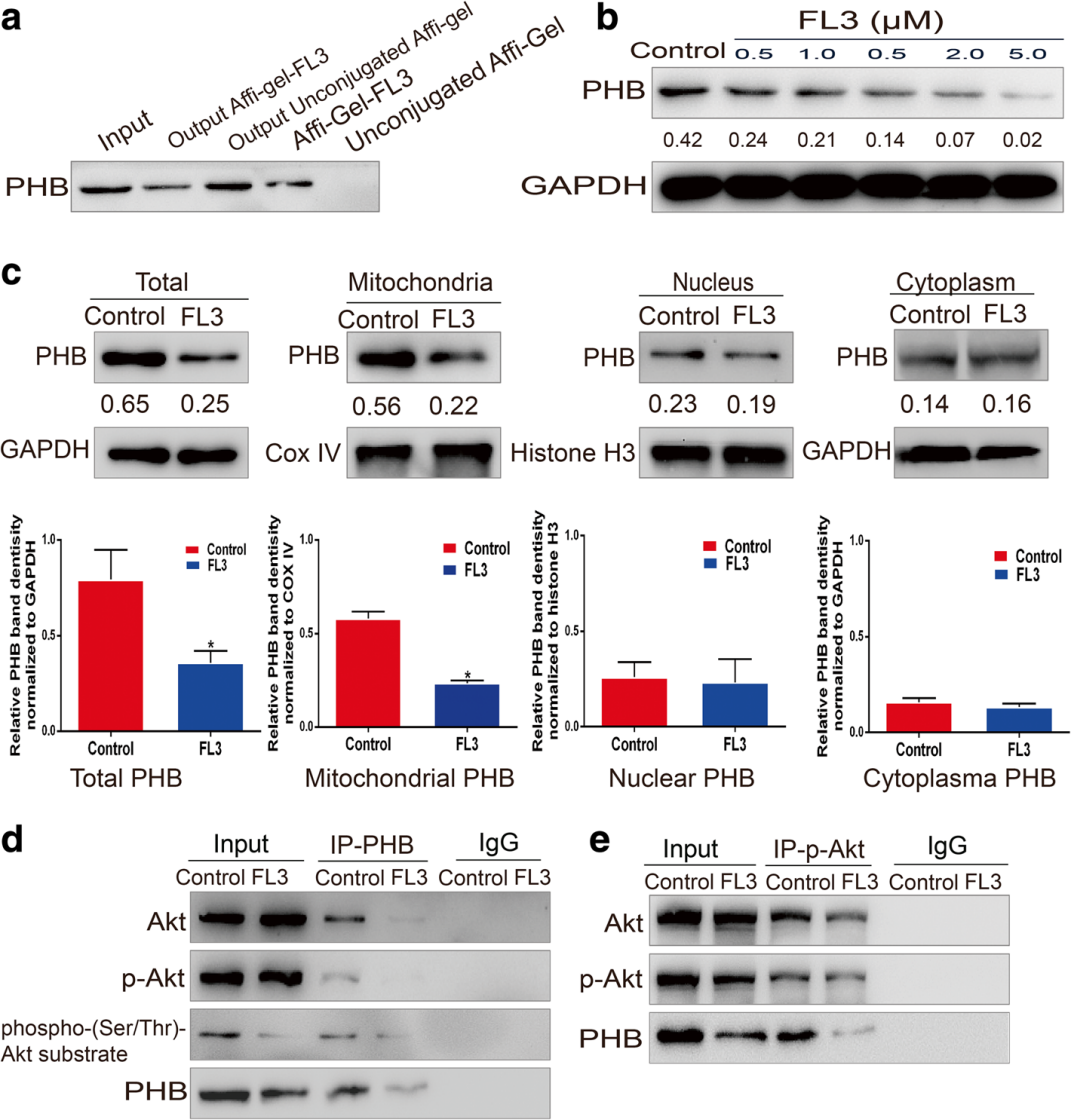
FL3 binds to PHB protein and inhibits the Akt/PHB interaction. **a** FL3-conjugated or unconjugated beads were incubated with total cell lysates from T24 cells. The eluted proteins were resolved on 10% SDS-PAGE gels for Western blotting with primary PHB antibody. **b** After incubation with indicated concentrations of FL3 for 24 h, total proteins of T24 cells were extracted and subjected to Western blot with primary PHB antibody. The Western blots have been quantified by densitometry and the quantitative values have been incorporated below the Western blot bands. **c** Subcellular fractions including cytoplasm, nucleus and mitochondria were isolated from the FL3-treated or control T24 cells. Then these fractions were lysed to obtain their total proteins, followed by Western blot analysis. Histograms show PHB protein intensity normalized to GAPDH, COX IV and Histone H3, respectively. The values represent the mean ± SD of three independent experiments. *P denotes < 0.05. The Western blots have been quantified by densitometry and the quantitative values have been incorporated below the Western blot bands. **d** After treatment with 0.5 μM FL3, total cell lysates of T24 cells were immunoprecipitated with primary PHB antibody. Then the eluted proteins were subjected to Western blot analysis with primary antibodies as indicated (left panel). **e** After treatment with 0.5 μM FL3, total cell lysates of T24 cells were immunoprecipitated with primary phospho-Akt (p-Akt) antibody. Then the eluted proteins were subjected to Western blot analysis with primary antibodies as indicated (left panel)

Because PHB is the direct target of Flavaglines in Jurkat T cells [12], FL3-Affigel-10 beads were used to determine whether FL3 targets PHB protein in UCB cell lines using immunoprecipitation assay. As shown in Fig. 2a, PHB protein was pulled down by the FL3-Affigel-10 beads, but there was no PHB binding to the unconjugated-Affigel-beads, indicating that FL3 binds to PHB protein.

### FL3 decreases PHB localization in the mitochondria

A recent study showed that FL3 treatment could influence the localization of PHB in cardiomyocytes by promoting PHB translocation from nucleus to mitochondria [13], however, it remains to be determined in UCB cells. We first measured the total PHB protein levels of FL3-treated T24 cells, and the result showed that the protein levels of PHB was slowly decreased by FL3 treatment (Fig. 2b). Although ubiquitously existed in multiple cellular compartments and each location stands for different functions, PHB mainly distributes in the mitochondria and participates in the stability of mitochondrial structure and functions [4, 7, 17–19]. We detected the change of PHB protein levels in main cellular fractions after FL3 treatment, and the results revealed that mitochondrial PHB levels was dramatically decreased, whereas nuclear PHB expression and cytoplasmic PHB expression were not significantly changed (Fig. 2c), suggesting that FL3 dose not promote PHB translocation from mitochondria to nucleus and cytoplasm.

### FL3 inhibits the Akt/PHB interaction and decreases PHB phosphorylation

PHB is a substrate of Akt, it is activated and transferred to the mitochondria after phosphorylation by Akt at Thr-258 site [11, 20]. FL3 might impede PHB localization within the mitochondria by blocking the interaction of PHB with Akt, which subsequently inhibits the phosphorylation process of PHB. To test this, total proteins of FL3-treated T24 cells were subjected to immunoprecipitation assay with primary PHB or phospho-Akt (Ser 473) antibody followed by western blot analysis. When proteins were immunoprecipitated with PHB antibody, FL3 treatment resulted in significant decrease of pulled-down PHB, Akt and phospho-Akt (p-Akt) compared to the control (Fig. 2d). If the proteins were immunoprecipitated with p-Akt antibody, pulled-down Akt and phospho-Akt levels were not significantly different, whereas the pulled-down PHB was significantly decreased compared to control (Fig. 2e).

Due to the shortage of available antibody recognizing phosphorylated PHB protein, we indirectly detected phosphorylated levels of PHB with a phospho-(Ser/Thr) Akt substrate (PAS) antibody, which can preferentially recognize peptides and proteins containing phospho-Ser/Thr preceded by Lys/Arg at positions − 5 and − 3 in a manner largely independent of other surrounding amino acids. As shown in Fig. 2d, a protein between 25 and 35 kDa was pulled down by PAS, and its expression was clearly reduced by FL3 compared to the control. This result indirectly showed that FL3 treatment decreased PHB phosphorylated levels.

Taken together, these data showed that FL3 inhibited the interaction of Akt and PHB, which in turn decreased PHB phosphorylation and localization in the mitochondria.

### FL3 does not induce cell apoptosis in UCB cells

A recent study has revealed the apoptotic activity of FL3 in certain cancer cells, such as HL60 and Hela cells, by triggering the translocation of Apoptosis Inducing Factor (AIF) and caspase-12 to the nucleus [16]. We also examined the apoptotic activity of FL3 in UCB T24 and BIU cells by flow cytometry assay. However, our results did not show a similar result that FL3 treatment did not significantly increase the apoptotic number of both T24 and BIU cells, suggesting that FL3 does not induce cell apoptosis in UCB cells (Additional file 1: Figure S1) .

### FL3 induces cell cycle arrest in the G2/M phase

**Fig. 3:**
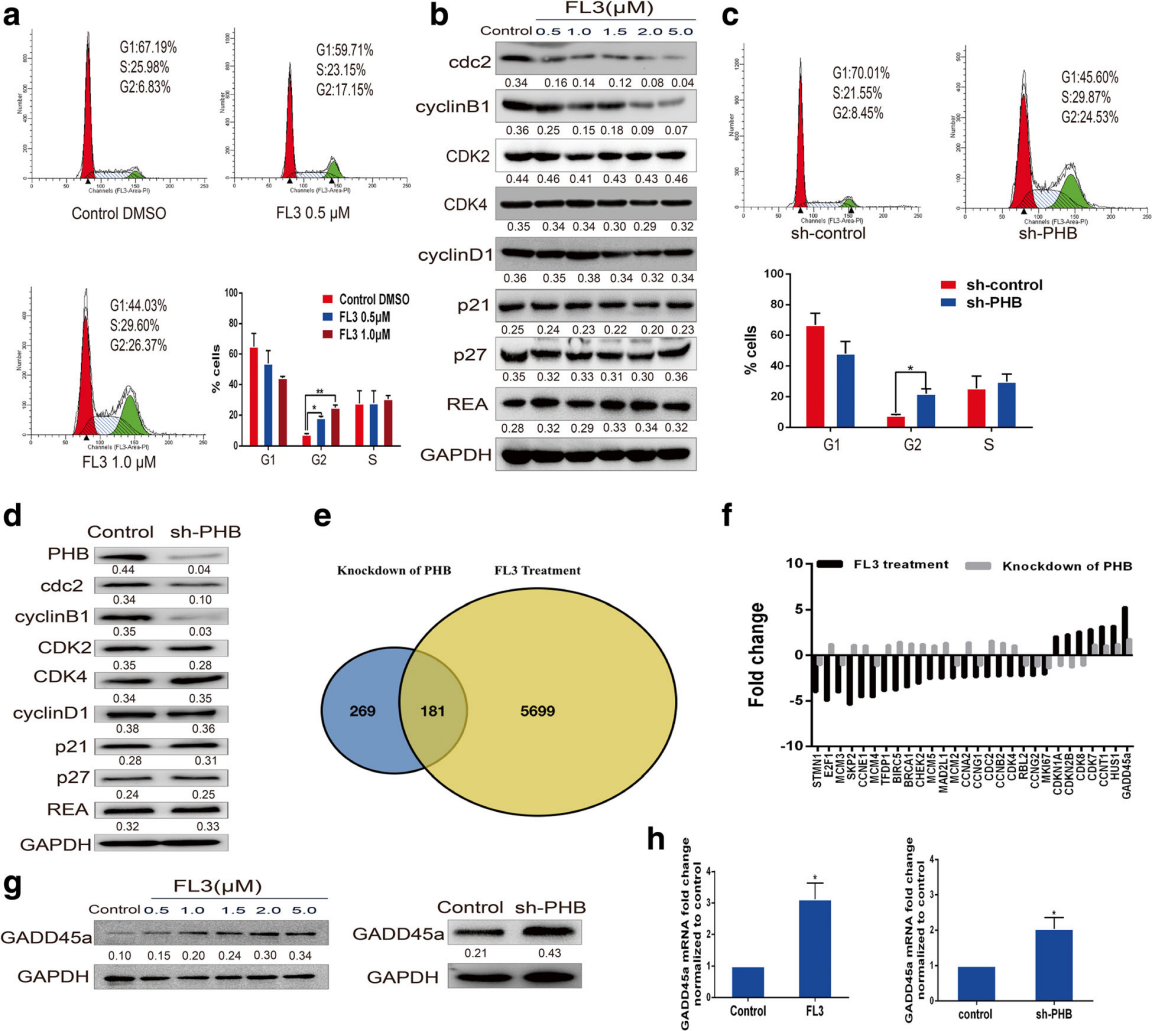
FL3-induced cell cycle inhibition in the G2/M phase via the GADD45α pathway. **a** After incubation with FL3 for 24 h, T24 cells were harvested and subjected to flow cytometry analysis. The percentage of cells in each phase was presented in the histograms, data represent mean ± SD of three independent experiments, *P < 0.05, **P < 0.01 means significance. **b** Total cell lysates were isolated from T24 cells treated with concentrations of FL3 for 24 h, and subjected to Western blot analysis with indicated antibodies (left panel). The Western blots have been quantified by densitometry and the quantitative values have been incorporated below the Western blot bands. **c** Cell cycle distribution of PHB knockdown T24 cells was analyzed by flow cytometry, and the percentage of cells in each phase was shown as histograms, data represent mean ± SD of three independent experiments, *P < 0.05 denotes a significant difference. **d** Total cell lysates from PHB knockdown or control T24 cells were isolated and subjected to Western blot analysis with the indicated antibodies (left panel). The Western blots have been quantified by densitometry and the quantitative values have been incorporated below the Western blot bands. **e** Regulated genes after FL3 treatment or PHB knockdown in T24 cells were compared with microarray analysis of mRNA expression. **f** Commonly regulated genes were analyzed in both microarray analyses, and GADD45α was significantly upregulated in both analyses. **g** Total cell lysates from FL3-treated or PHB knockdown T24 cells were subjected to Western blot analysis with primary GADD45α antibody, indicating that the protein levels of GADD45α were upregulated. **h** Quantitative real-time PCR (qRT-PCR) was performed to detect the mRNA expression of GADD45α. *P < 0.05 denotes significant difference compared to control

It has been shown that FL3 affects cell cycle distribution in Jurkat T cells [12], so we determined whether it has similar effects in UCB cells. As Fig. 3a shown, FL3 resulted in cell cycle arrest in the G2/M phase by increasing the percentage of cells in the G2 phase from 6.83% in control cells to 17.15% and 26.37% in FL3 -treated T24 cells, and the percentage of cells in S phase nonsignificantly ranged from 25.98% to 23.15% and 29.60%. In addition, protein expressions of cdc2 and cyclin B1, which are implicated with G2-M transition [21], were dramatically decreased (Fig. 3b). In contrast, protein expressions of G1-S transition related cyclins such as CDK2, CDK4, and cyclin D1 were unchanged. Also, several key regulators of cell cycle including p21, p27 and REA (repressor of estrogen receptor activity) [22–24] were also analyzed, the results showed that their protein expressions were not significantly changed after FL3 treatment (Fig. 3b).

### PHB knockdown mimics the inhibitory effect of FL3 on the cell cycle progression in UCB T24 cells

To clarify the action of FL3 on PHB signaling, we knocked down PHB expression in T24 cells to determine whether PHB knockdown would mimic the inhibitory effect of FL3 on cell cycle progression. Flow cytometry assay was performed and the results showed that PHB knockdown increased the percentage of cells in the G2 phase from 8.45% to 24.53%, accompanying with slight and insignificant increase of cells in the S phage from 21.55% to 29.87% (Fig. 3c). Meanwhile, the protein levels of cdc2 and cyclin B1 were decreased, whereas that of CDK2, CDK4, cyclin D1, p21, p27 and REA were not significantly changed (Fig. 3d). These data suggests that knockdown of PHB mimics the inhibitory effect of FL3 on cell cycle progression.

### GADD45α is upregulated after FL3 treatment and PHB knockdown

To identify the common pathways regulated by FL3 treatment or PHB knockdown, microarray analyses of mRNA expression were performed in FL3-treated or PHB knockdown T24 cells. Totally regulated genes were compared and presented as shown in Fig. 5e, a total of 181 genes were commonly regulated in both analyses. Since FL3 and PHB knockdown inhibits cell cycle progression in the G2/M phase, we tried to explore the underlying mechanism of this effect. A total of 84 cell cycle-related genes [25] were compared with each other in both analyses. Significantly regulated genes (more than 1.7-fold) were selected out in Additional file 2: Table S1, 28 genes were significantly regulated by FL3. Comparing the 28 genes in microarray analysis of PHB knockdown cells, 11 genes were commonly regulated by FL3 and PHB knockdown (Fig. 3f), among of which GADD45α (the growth arrest and DNA damage-inducible alpha), a checkpoint gene during the G2-M transition [26], was verified as the only significantly regulated gene (5.26 vs. 1.76-fold). Upregulation of GADD45α was further validated by western blotting and quantitative real time-PCR (qRT-PCR) (Fig. 3g-h). The results indicated that FL3-induced cell cycle arrest might be GADD45α-dependent.

### FL3-mediated cell cycle inhibition is GADD45α-dependent

**Fig. 4:**
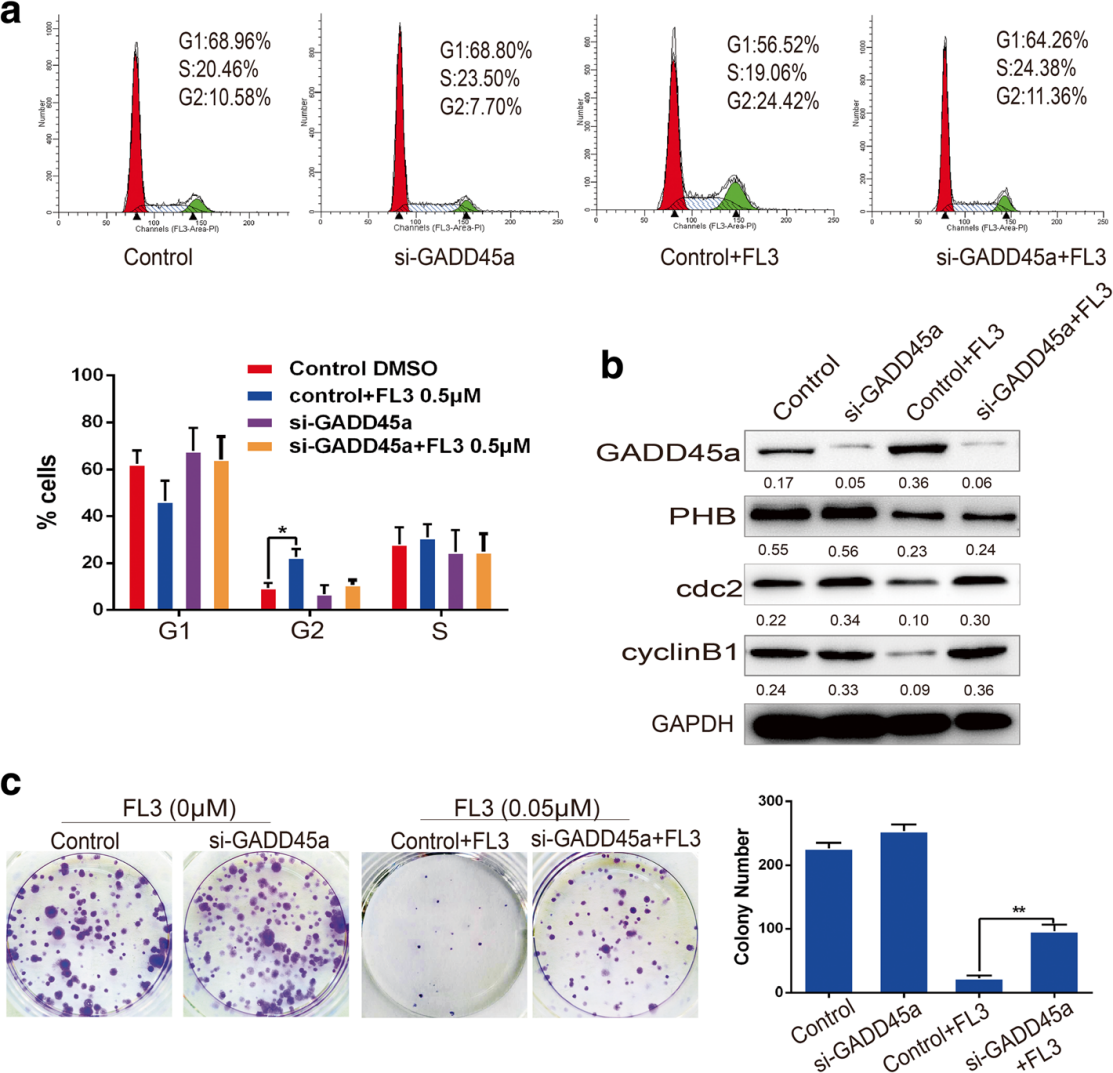
Repression of GADD45α decreases the inhibitory effect of FL3 on cell cycle progression. **a** Flow cytometry assay was performed in T24 cells to measure the effect of FL3 on the cell cycle distribution in the presence or absence of siGADD45α-RNA. The percentage of cells in each phase was shown in the histograms; data represent the mean ± SD of three independent experiments, *P < 0.05 indicates a significant difference. **b** Total cell lysates from indicated T24 cells (up panel) were harvested and subjected to Western blot analysis with the indicated proteins (left panel). **c** Cell colony formation experiments were performed to measure the inhibitory effect of FL3 on cell proliferation of T24 cells in the presence or absence of GADD45α. Histograms represent the mean ± SD numbers of colonies of three independent experiments. **P < 0.01 indicates significance

To determine whether FL3-induced cell cycle arrest is GADD45α-dependent, a rescue experiment was performed by repressing GADD45α expression with siRNA in T24 cells. Flow cytometry assay demonstrated that FL3 treatment did not significantly change the percentage of cells in the G2 phase (from 7.70% to 11.36%) in GADD45α-silenced cells, whereas the percentage of cells in this phase increased from 10.58% to 24.42% in the control cells (Fig. 4a, b). Thus, repression of GADD45α rescued the inhibitory effect of FL3 on cell cycle progression. In addition, repression of GADD45α did not change the protein levels of PHB, cdc2 and cyclinB1 (Fig. 4c). Moreover, repression of GADD45α attenuated the inhibitory effect of FL3 on the colony formation ability of T24 cells (Fig. 4d), indicating that GADD45α is critical for UCB cell proliferation. Taken together, FL3-induced cell cycle inhibition is GADD45α-dependent.

### FL3 suppresses xenograft tumor growth in vivo

**Fig. 5:**
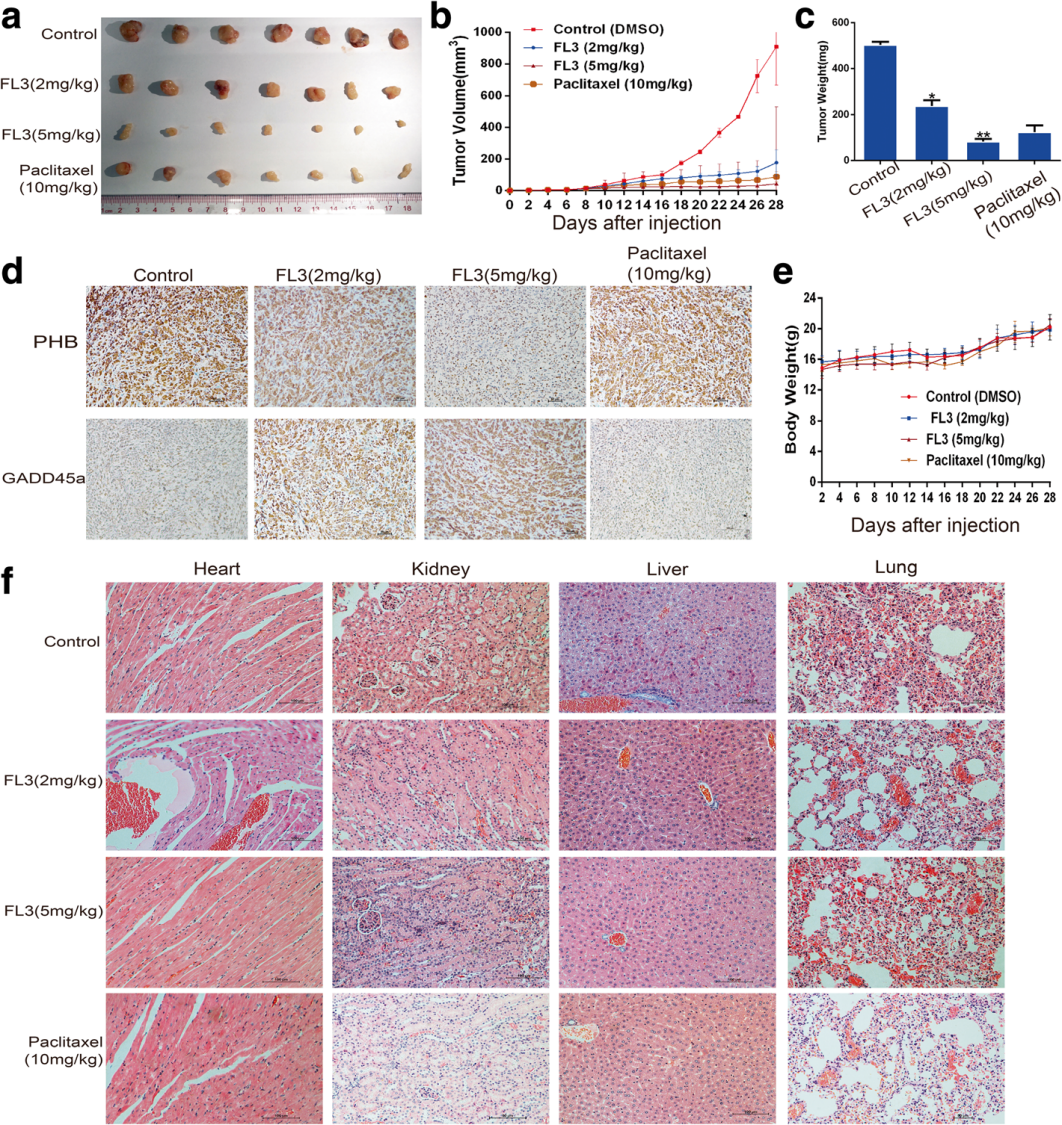
FL3 inhibits growth of UCB tumor xenografts in vivo. **a** The xenograft tumors were isolated from mice at the end of study. **b** Tumor volumes were recorded from the date of injection to the end of the study (mean, *n* = 7). **c** Histograms present the mean tumor weight in each group, means ± SD (*n* = 7). **P < 0.01, ***P < 0.001 indicates a significant difference between FL3-treated mice and control mice. **d** Tumors were embedded in paraffin and 5 μm thick sections were used for immunohistochemistry analysis with PHB or GADD45α antibody. **e** Body weights of mice were recorded along with the records of tumor volumes as dashed lines (mean, *n* = 7). **f** Main organs including heart, kidney, liver, and lung were removed from mice and embedded in paraffin for further hematoxylin eosin staining

To further verify the inhibitory role of FL3 on cell proliferation in vivo, xenograft tumor models were constructed (Fig. 5a). Both FL3 and paclitaxel treatments significantly inhibited the volumes and weights of tumors compared to the control (Fig. 5b, c). Furthermore, immunohistochemistry assay presented that FL3 treatment resulted in slightly decreased expression of PHB protein compared to the control (Fig. 5d). In contrast, the expression of GADD45α was slightly elevated. Meaningfully, there was no significant body weight loss in FL3-treated mice compared to the control (Fig. 5e). H&E staining of the livers, lungs, hearts, and kidneys showed no major organ-related toxicities from FL3 treatment compared to the control group (Fig. 5f).

## Discussion

Current chemotherapeutic drugs have limited effectiveness and intolerable toxicities in most cases of UCB. Therefore, there is an urgent need for developing novel therapeutic modalities of UCB. It has been widely reported that PHB is critical for many cellular biologic responses including senescence, development and tumorigenesis [4, 7, 16, 27–30]. Our previous study revealed that in UCBs, PHB is frequently overexpressed in > 80% clinical patients, and UCB with high expression of PHB predicts a poor disease-free survival of the patient, suggesting a potential therapeutic target of PHB in UCB [11].

Recent studies have shown that flavaglines can directly binds to PHB to inhibit the activation of PHB-mediated signaling pathways, consequently leading to inhibition of protein synthesis, cell cycle progression, and cell growth in cancer cells [12, 31, 32]. Meanwhile, flavaglines present little cytotoxicity to healthy cells at nanamolar concentrations [13, 16, 33, 34]. Therefore, the discovery of flavaglines brings new hope for the treatment of UCB. In our present study, we have demonstrated that FL3, a derivative of flavaglines, directly targets PHB and inhibits UCB cell proliferation both in vitro and in vivo.

The PI3K/Akt pathway has been implicated in many cellular biological responses and the development of carcinogenesis [35–38]. We found that both Akt and PHB were upregulated in UCB tissues and correlated with the poor prognosis of UCB patients [11]. Recent studies have demonstrated that PHB is a substrate of Akt, and Akt-mediated phosphorylation is required for PHB localization within the mitochondria as well as UCB cell proliferation [11, 39]. Mitochondrial PHB is closely associated with mitochondrial functions including mitochondrial stabilization, resistance of apoptosis, the respiratory chain, and components of mitochondrial DNA [40, 41]. Correlating with cell proliferation, reduction of PHB expression in the mitochondria might lead to mitochondria-related apoptosis and cell cycle inhibition. In this study, our results showed that FL3 treatment decreased the interaction of Akt and PHB as well as PHB localization within mitochondria. However, we did not observe the similar effect of inducing apoptosis in UCB cells as FL3 dose in HL60 and Hela cells. We thought that the functions of FL3 to induce cellular apoptosis might be tumor type-specific.

Since the activity of FL3 is based on its binding with PHB protein, knockdown of PHB could mimic the inhibitory effect of FL3 on UCB cell cycle progression. We found that the expression of GADD45α, a regulator of cell cycle, was significantly upregulated after either FL3 treatment or PHB knockdown. *GADD45α* is a member of the growth arrest and DNA damage 45 (*GADD45*) gene family, which encodes three highly homologous proteins GADD45α, GADD45β, and GADD45γ [37]. GADD45α localizes to the nucleus and involves in the inhibition of cell cycle G2-M transition by inhibiting the activation of cdc2/cyclin B1 kinases, leading to the initiation of the G2/M checkpoint mechanism and subsequently arrests cell cycle progression in the G2/M phase [21, 26, 42, 43]. Consistent with theses studies, the expression of GADD45α expression was upregulated while the expression of cdc2 and cyclin B1 were decreased after FL3 treatment in UCB cells. If the expression of GADD45α is repressed, the inhibitory effect of FL3 on cell cycle would be rescued. Thus, our results has strongly suggested that FL3-induced G2/M cell cycle inhibition is GADD45α-dependent.

GADD45α involved cell cycle regulation is controlled by Akt/FOXO3A pathway by that Akt represses the activity of GADD45α and promotes cell cycle progression [44]. In our results, the expression of GADD45α is also controlled by PHB. Taking together, GADD45α expression is negatively regulated by Akt/PHB. By directly targeting with PHB protein, FL3 blocks the interaction of Akt and PHB and in turn attenuates the control of Akt/PHB to GADD45α, with a consequence of upregulation of GADD45α and cell cycle inhibition in the G2/M phase.

## Conclusions

**Fig. 6:**
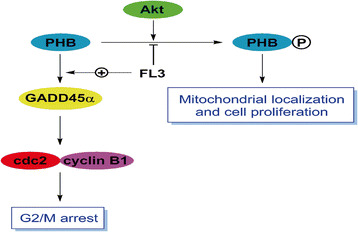
Line map of the potential mechanisms that FL3 inhibits UCB cell cycle progression and cells proliferation. FL3 blocks the interaction of Akt and PHB, which in turn inhibits the process of PHB phosphorylation by Akt and decreases the PHB localization within mitochondria, leading to inhibition of UCB cell proliferation. Meanwhile, FL3 treatment attenuates the control of PHB to GADD45α, which consequently activates the GADD45α-dependent cell cycle arrest in the G2/M. Taken together, FL3 inhibits the UCB cell proliferation

This study provides data to elucidate the mechanism of FL3 inhibiting UCB cell proliferation (Fig. 6), suggesting that FL3 might be a therapeutic agent for the treatment of UCB in the future.

All data in this study have been recorded at Sun Yat-sen University Cancer Center (Number RDDB2017000176).

## Additional files

Additional file 1: Figure S1. FL3 does not induce apoptosis in UCB T24 and BIU cells. After incubation with 0.5 μM and 1.0 μM FL3 for 24 h, T24 and BIU cells were harvested and stained with Annexin V-FITC/PE to count the numbers of apoptotic cells. Q3–1, dead cells; Q3–2, late apoptotic cells; Q3–3, live cells; Q3–4, early apoptotic cells. This experiment was independently repeated for three times. Histograms display the number of cells as the mean ± SD of three independent experiments. (TIFF 67307 kb)

Additional file 2: Table S1. List of cell cycle-related genes regulated by FL3 treatment or PHB knockdown in T24 cells using microarray analysis of mRNA expression. (DOC 94 kb)
