# Correction to: Flavagline analog FL3 induces cell cycle arrest in urothelial carcinoma cell of the bladder by inhibiting the Akt/PHB interaction to activate the GADD45α pathway

**DOI:** 10.1186/s13046-021-01923-9

**Published:** 2021-03-30

**Authors:** Gangjun Yuan, Xin Chen, Zhuowei Liu, Wensu Wei, Qinghai Shu, Hussein Abou-Hamdan, Lijuan Jiang, Xiangdong Li, Rixin Chen, Laurent Désaubry, Fangjian Zhou, Dan Xie

**Affiliations:** 1 State Key Laboratory of Oncology in South China; Collaborative Innovation Center for Cancer Medicine, Sun Yat-sen University Cancer Center, Guangzhou, 510060 China; 2 Department of Urology, Sun Yat-sen University Cancer Center, Guangzhou, China; 3 School of Material Science and Engineering, Beijing Institute of Technology, Beijing, China; 4 Therapeutic Innovation Laboratory, UMR7200, CNRS/University of Strasbourg, Strasbourg, France; 5 Sino-French Joint Lab of Food Nutrition/Safety and Medicinal Chemistry, College of Biotechnology, Tianjin University of Science and Technology, Tianjin, China

**Correction to: J Exp Clin Cancer Res 37, 21 (2018)**

**https://doi.org/10.1186/s13046-018-0695-5**

Following publication of the original article [1], the authors identified minor errors in image-typesetting in Fig. 4; specifically in Fig. 4c.

**Fig. 4:**
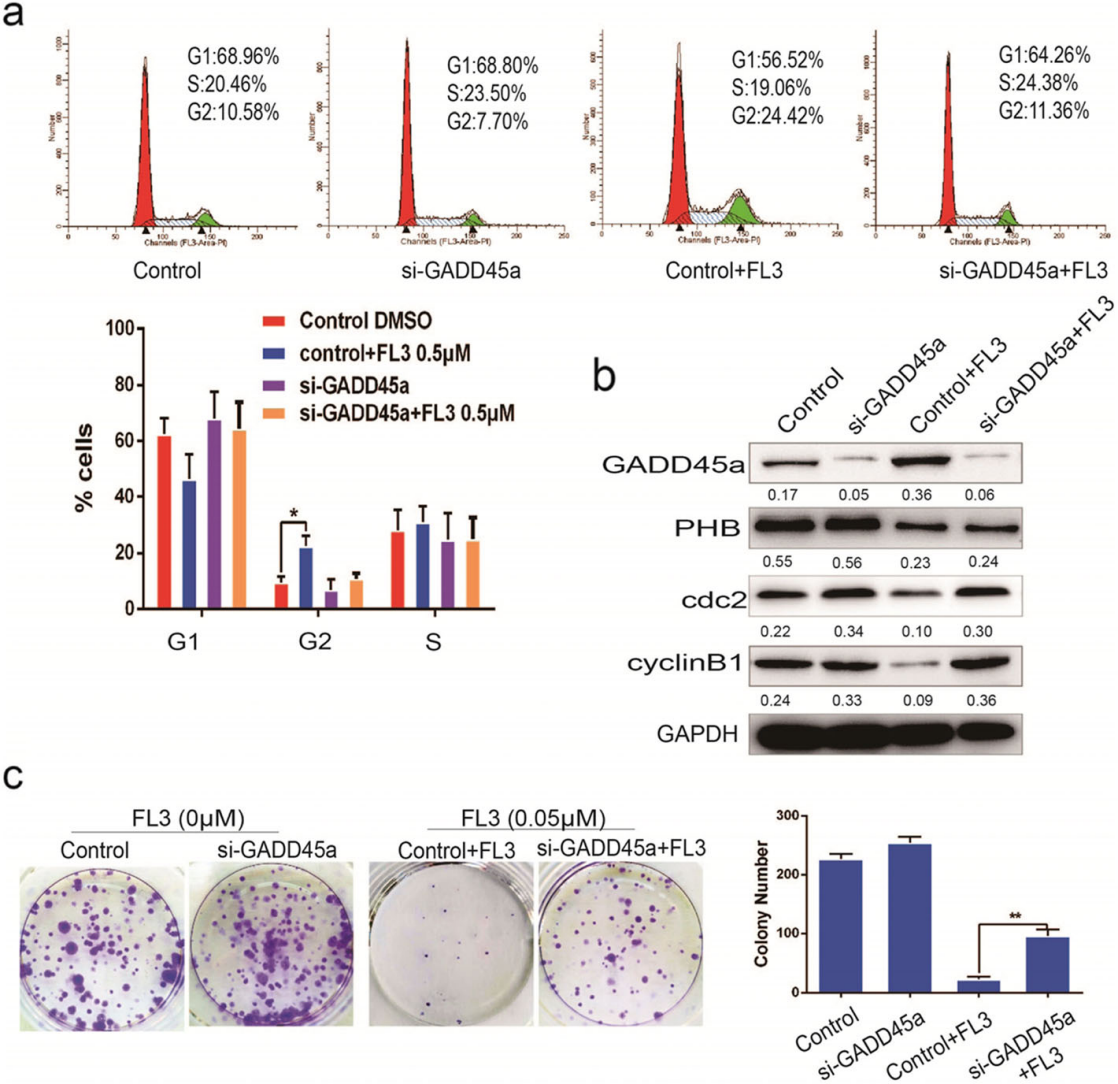
Repression of GADD45α decreases the inhibitory effect of FL3 on cell cycle progression. **a** Flow cytometry assay was performed in T24 cells to measure the effect of FL3 on the cell cycle distribution in the presence or absence of siGADD45α-RNA. The percentage of cells in each phase was shown in the histograms; data represent the mean ± SD of three independent experiments, *P < 0.05 indicates a significant difference. **b** Total cell lysates from indicated T24 cells (up panel) were harvested and subjected to Western blot analysis with the indicated proteins (left panel). **c** Cell colony formation experiments were performed to measure the inhibitory effect of FL3 on cell proliferation of T24 cells in the presence or absence of GADD45α. Histograms represent the mean ± SD numbers of colonies of three independent experiments. **P < 0.01 indicates significance

The corrected figure is given below. The correction does not have any effect on the results or conclusions of the paper.

## References

[1] Yuan G, Chen X, Liu Z, et al. Flavagline analog FL3 induces cell cycle arrest in urothelial carcinoma cell of the bladder by inhibiting the Akt/PHB interaction to activate the GADD45α pathway. J Exp Clin Cancer Res. 2018;37:21 https://doi.org/10.1186/s13046-018-0695-5. 10.1186/s13046-018-0695-5 PMC5804081 29415747

