# Correction: Flavagline analog FL3 induces cell cycle arrest in urothelial carcinoma cell of the bladder by inhibiting the Akt/PHB interaction to activate the GADD45α pathway

**DOI:** 10.1186/s13046-022-02502-2

**Published:** 2022-09-30

**Authors:** Gangjun Yuan, Xin Chen, Zhuowei Liu, Wensu Wei, Qinghai Shu, Hussein Abou-Hamdan, Lijuan Jiang, Xiangdong Li, Rixin Chen, Laurent Désaubry, Fangjian Zhou, Dan Xie

**Affiliations:** 1 State Key Laboratory of Oncology in South China; Collaborative Innovation Center for Cancer Medicine, Sun Yat-sen University Cancer Center, Guangzhou, 510060 China; 2 Department of Urology, Sun Yat-sen University Cancer Center, Guangzhou, China; 3 School of Material Science and Engineering, Beijing Institute of Technology, Beijing, China; 4 Therapeutic Innovation Laboratory, UMR7200, CNRS/University of Strasbourg, Strasbourg, France; 5 Sino-French Joint Lab of Food Nutrition/Safety and Medicinal Chemistry, College of Biotechnology, Tianjin University of Science and Technology, Tianjin, China

**Correction: J Exp Clin Cancer Res 37, 21 (2018)**

**https://doi.org/10.1186/s13046-018-0695-5**

Following publication of the original article [1], an error was identified in the image of PHB Control and Paclitaxel (10 mg/kg) in Fig. 5d.

**Fig. 5:**
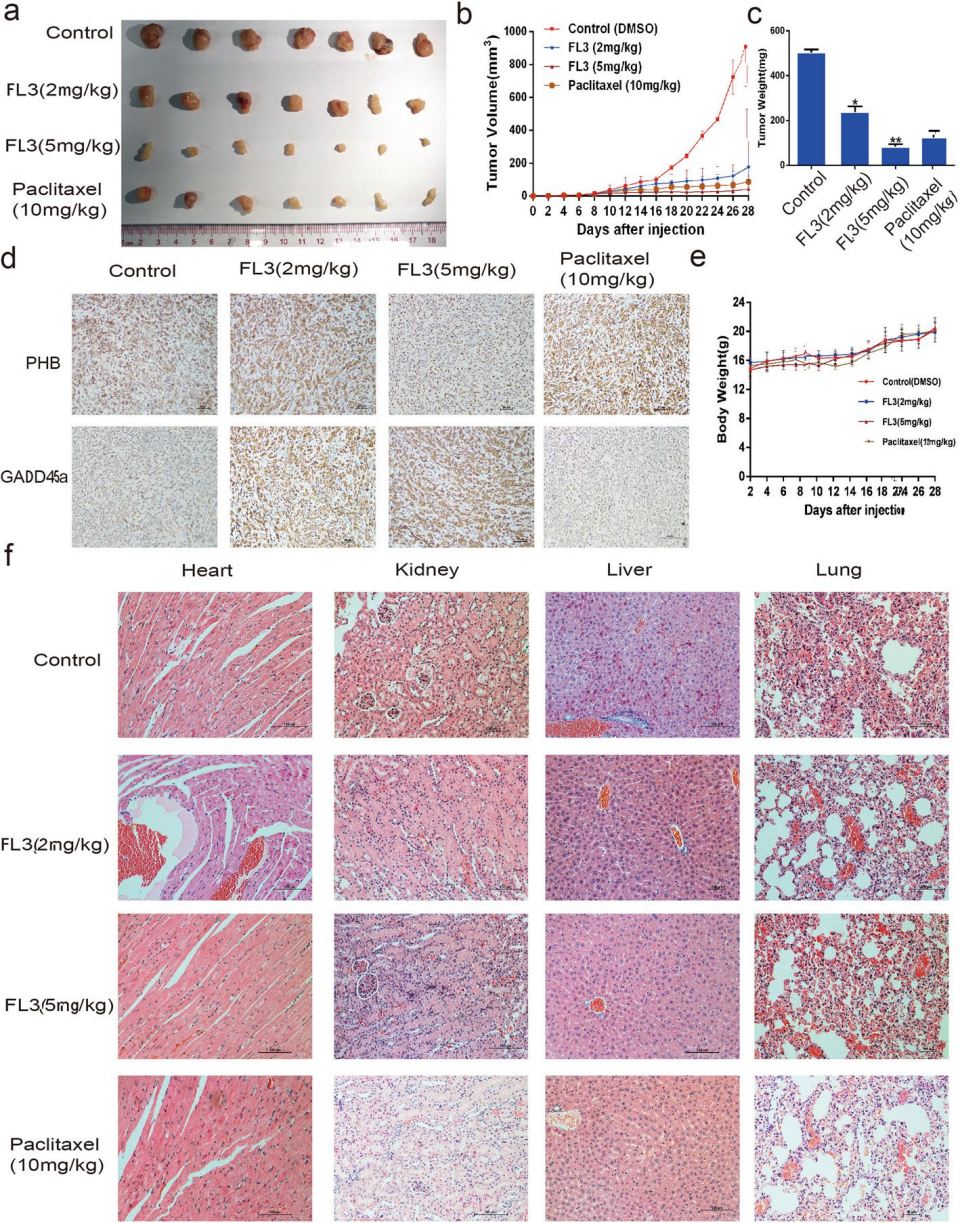
FL3 inhibits growth of UCB tumor xenografts in vivo. **a** The xenograft tumors were isolated from mice at the end of study. **b** Tumor volumes were recorded from the date of injection to the end of the study (mean, *n* = 7). **c** Histograms present the mean tumor weight in each group, means ± SD (*n* = 7). **P < 0.01, ***P < 0.001 indicates a significant difference between FL3-treated mice and control mice. **d** Tumors were embedded in paraffin and 5 μm thick sections were used for immunohistochemistry analysis with PHB or GADD45α antibody. **e** Body weights of mice were recorded along with the records of tumor volumes as dashed lines (mean, *n* = 7). **f** Main organs including heart, kidney, liver, and lung were removed from mice and embedded in paraffin for further hematoxylin eosin staining

The correction does not have any effect on the results or conclusions of the paper. The original article has been corrected.
