# Supplementary material for: Flavagline analog FL3 induces cell cycle arrest in urothelial carcinoma cell of the bladder by inhibiting the Akt/PHB interaction to activate the GADD45α pathway
Source: J Exp Clin Cancer Res. 2018 Feb 7;37:21. doi: 10.1186/s13046-018-0695-5 (PMC5804081; doi:10.1186/s13046-018-0695-5)
Supplement: Supplementary file 2 — List of cell cycle-related genes regulated by FL3 treatment or PHB knockdown in T24 cells using microarray analysis of mRNA expression. (DOC 94 kb) [file 13046_2018_695_MOESM2_ESM.doc]

Table S1. List of cell cycle-related genes regulated by FL3 treatment or PHB knockdown in T24 cells using microarray analysis of mRNA expression.

| Gene | Fold Change | | Function |
| --- | --- | --- | --- |
|  | FL3 Treatment | PHB Knockdown |  |
| ABL1 | 1.06 | 1.02 | S phase and DNA replication; regulation of cell cycle |
| ANAPC2 | 1.13 | 1.01 | G1 phase and G1/S transition;G2 phase and G2/M transition; regulation of cell cycle |
| ATM | 1.01 | 1.01 | Cell cycle checkpoint and cell cycle arrest; negative regulation of cell cycle |
| ATR | 1.03 | 1.00 | Cell cycle checkpoint and cell cycle arrest; regulation of cell cycle |
| AURKA | -1.32 | 1.19 | regulation of cell cycle |
| AURKB | -1.52 | 1.48 | M phase |
| RBBP8 | -1.23 | 1.64 | Cell cycle checkpoint and cell cycle arrest |
| BCCIP | 1.16 | 1.15 | G2 phase and G2/M transition; regulation of cell cycle |
| BCL2 | 1.12 | 1.04 | regulation of cell cycle |
| BIRC5 | -3.82 | 1.26 | G2 phase and G2/M transition |
| BRCA1 | -3.5 | 1.32 | Cell cycle checkpoint and cell cycle arrest; negative regulation of cell cycle |
| BRCA2 | -1.1 | 1.31 | Cell cycle checkpoint and cell cycle arrest; regulation of cell cycle |
| CASP3 | 1.22 | 1.12 | Cell cycle checkpoint and cell cycle arrest; negative regulation of cell cycle |
| CCNA2 | -2.4 | 1.41 | G2 phase and G2/M transition; cell cycle checkpoint and cell cycle arrest |
| CCNB1 | -1.68 | 1.54 | G2 phase and G2/M transition; regulation of cell cycle |
| CCNB2 | -2.28 | 1.32 | M phase; regulation of cell cycle |
| CCNC | -1.02 | 1.08 | regulation of cell cycle |
| CCND1 | 1.35 | 1.03 | G1 phase and G1/S transition; regulation of cell cycle |
| CCND2 | -1.1 | 1.13 | regulation of cell cycle |
| CCND3 | -1.91 | 1.13 | regulation of cell cycle |
| CCNE1 | -4.58 | 1.01 | G1 phase and G1/S transition; regulation of cell cycle |
| CCNF | 1.01 | 1.11 | M phase; regulation of cell cycle |
| CCNG1 | -2.38 | -1.13 | G2 phase and G2/M transition |
| CCNG2 | -2.24 | -1.19 | cell cycle checkpoint and cell cycle arrest |
| CCNH | 1.36 | 1.34 | G2 phase and G2/M transition; regulation of cell cycle |
| CCNT1 | 3.14 | 1.06 | G2 phase and G2/M transition; regulation of cell cycle |
| CDC16 | 1.09 | 1.11 | regulation of cell cycle |
| CDC2 | -2.35 | 1.55 | cell cycle checkpoint and cell cycle arrest; regulation of cell cycle |
| CDC20 | -1.16 | 1.47 | regulation of cell cycle |
| CDC25A | 1.27 | 1.05 | G1 phase and G1/S transition; G2 phase and G2/M transition; cell cycle checkpoint and cell cycle arrest |
| CDC25C | -1.92 | 1.19 | M phase; cell cycle checkpoint and cell cycle arrest |
| CDC34 | 1.93 | 1.02 | G1 phase and G1/S transition; cell cycle checkpoint and cell cycle arrest |
| CDC6 | -1.19 | 1.76 | S phase and DNA replication; M phase; regulation of cell cycle |
| CDK2 | -1.36 | 1.36 | cell cycle checkpoint and cell cycle arrest; regulation of cell cycle |
| CDK4 | -2.25 | 1.05 | G1 phase and G1/S transition; regulation of cell cycle |
| CDK5R1 | 1.21 | 1.07 | G2 phase and G2/M transition; regulation of cell cycle |
| CDK5RAP1 | 1.01 | 1.46 | G2 phase and G2/M transition |
| CDK6 | 1.73 | -1.02 | G1 phase and G1/S transition; regulation of cell cycle |
| CDK7 | 2.82 | 1.13 | G2 phase and G2/M transition; regulation of cell cycle |
| CDK8 | 2.57 | -1.18 | regulation of cell cycle |
| CDKN1A | 2.04 | -1.11 | cell cycle checkpoint and cell cycle arrest; regulation of cell cycle |
| CDKN1B | 1.99 | -1.23 | G1 phase and G1/S transition; cell cycle checkpoint and cell cycle arrest |
| CDKN2A | -1.3 | -1.27 | cell cycle checkpoint and cell cycle arrest |
| CDKN2B | 2.25 | -1.09 | cell cycle checkpoint and cell cycle arrest; negative regulation of cell cycle |
| CDKN3 | -1.51 | -1.39 | G1 phase and G1/S transition; G2 phase and G2/M transition; cell cycle checkpoint and cell cycle arrest |
| CHEK1 | 1.29 | 1.03 | cell cycle checkpoint and cell cycle arrest |
| CHEK2 | -3.07 | 1.68 | cell cycle checkpoint and cell cycle arrest |
| CKS1B | -1.21 | 1.13 | G2 phase and G2/M transition; regulation of cell cycle |
| CKS2 | 1.58 | 1.22 | G2 phase and G2/M transition |
| CUL1 | 1.68 | 1.50 | G1 phase and G1/S transition; cell cycle checkpoint and cell cycle arrest |
| CUL2 | -1.25 | 1.33 | G1 phase and G1/S transition; cell cycle checkpoint and cell cycle arrest |
| CUL3 | 1.46 | 1.04 | G1 phase and G1/S transition; cell cycle checkpoint and cell cycle arrest |
| E2F1 | -4.97 | 1.02 | G1 phase and G1/S transition; regulation of cell cycle |
| E2F4 | 1.96 | 1.03 | regulation of cell cycle |
| GADD45a | 5.26 | 1.76 | cell cycle checkpoint and cell cycle arrest; regulation of cell cycle; G2 phase and G2/M transition |
| GTSE1 | -1.91 | 1.20 | G2 phase and G2/M transition n |
| HUS1 | 3.21 | 1.76 | cell cycle checkpoint and cell cycle arrest |
| KNTC1 | -1.99 | 1.25 | cell cycle checkpoint and cell cycle arrest; regulation of cell cycle |
| KPNA2 | 1.3 | 1.12 | G2 phase and G2/M transition |
| MAD2L1 | -2.53 | 1.05 | cell cycle checkpoint and cell cycle arrest |
| MAD2L2 | -1 | 1.38 | cell cycle checkpoint and cell cycle arrest |
| MCM2 | -2.49 | 1.19 | S phase and DNA replication; M phase |
| MCM3 | -4.04 | -1.11 | S phase and DNA replication; M phase |
| MCM4 | -4.58 | -1.05 | S phase and DNA replication; M phase |
| MCM5 | -2.56 | 1.16 | S phase and DNA replication; M phase |
| MDM2 | -1.33 | 1.04 | cell cycle checkpoint and cell cycle arrest |
| MKI67 | -2.05 | -1.13 | regulation of cell cycle |
| MNAT1 | 1.16 | 1.48 | G2 phase and G2/M transition |
| MRE1A | -1.23 | 1.22 | G2 phase and G2/M transition |
| NBN | 1.03 | 1.10 | cell cycle checkpoint and cell cycle arrest |
| RAD1 | 1.46 | 1.29 | cell cycle checkpoint and cell cycle arrest |
| RAD17 | 1.88 | 1.08 | cell cycle checkpoint and cell cycle arrest |
| RAD51 | -1.41 | 1.53 | cell cycle checkpoint and cell cycle arrest |
| RAD9A | -1.04 | -1.03 | cell cycle checkpoint and cell cycle arrest; regulation of cell cycle |
| RB1 | 1.85 | 1.23 | cell cycle checkpoint and cell cycle arrest; regulation of cell cycle |
| RBL1 | -1.6 | 1.33 | negative regulation of cell cycle |
| RBL2 | -2.25 | -1.10 | negative regulation of cell cycle |
| SERTAD1 | 4.26 | 1.30 | G2 phase and G2/M transition |
| SKP2 | -5.4 | 1.11 | G1 phase and G1/S transition; regulation of cell cycle |
| STMN1 | -4.01 | -1.06 | M phase |
| TFDP1 | -3.88 | 1.04 | regulation of cell cycle |
| TFDP2 | -1.18 | 1.06 | regulation of cell cycle |
| TP53 | 1.14 | 1.1 | cell cycle checkpoint and cell cycle arrest; negative regulation of cell cycle |
| WEE1 | 1.21 | 1.03 | S phase and DNA replication; M phase; cell cycle checkpoint and cell cycle arrest; regulation of cell cycle |
